# Characterization of temperature-sensitive alleles of the septation initiation network protein Mob1 in *Schizosaccharomyces pombe*

**DOI:** 10.17912/micropub.biology.001595

**Published:** 2025-04-28

**Authors:** Sarah M. Hanna, Alaina H. Willet, Kathleen L. Gould

**Affiliations:** 1 Department of Cell and Developmental Biology, Vanderbilt University School of Medicine, Nashville, TN, US

## Abstract

*Schizosaccharomyces pombe* Mob1 is the regulatory subunit of the protein kinase Sid2 . The Sid2- Mob1 complex is the most downstream acting component of the septation initiation network (SIN). In the absence of functional Mob1 , cells fail cytokinesis and become multinucleate. Here we characterize a set of temperature-sensitive *mob1* alleles by identifying the mutations within each allele, characterizing the extent of their growth defects, and visualizing the cell defects. Based on structural modeling, we hypothesize that the Mob1 mutations interfere with Mob1 stability and its ability to bind the N-terminal regulatory region of Sid2 .

**Figure 1. Characterization and comparison of mob1 alleles.**
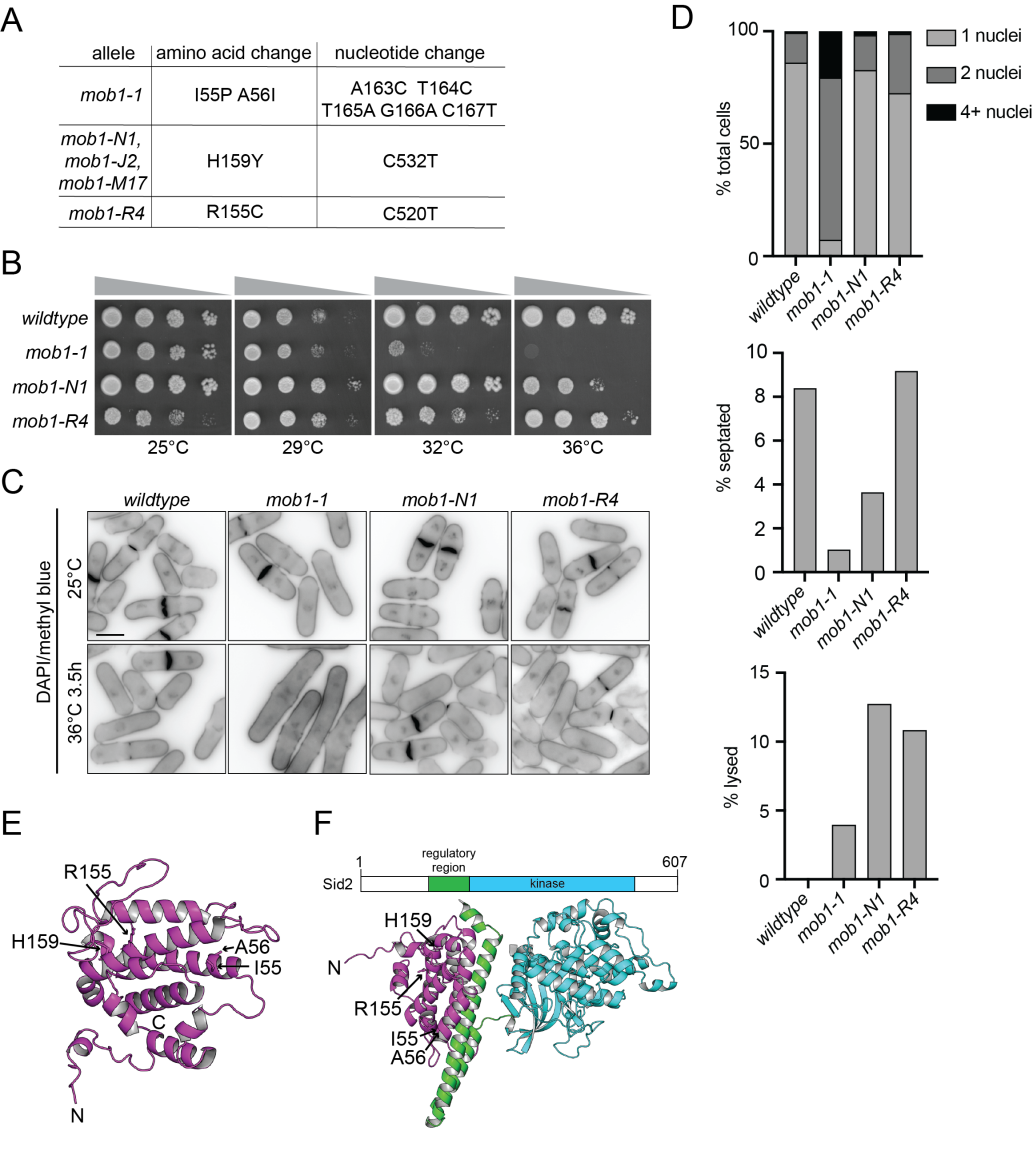
(A) The mutations encoded by each *mob1* allele are listed. (B) The indicated strains were grown in liquid YE media at 25°C until they reached mid-log phase and then adjusted to the same cell concentrations measured by optical density (Moreno et al., 1991). Next, 10-fold serial dilutions were made and 2.5 µL of each was spotted on YE agar plates and incubated at the indicated temperatures for 2-3 days prior to imaging. The spot assays were done twice and a representative is shown. (C) The indicated strains were grown in liquid YE media at 25˚C. Samples were collected before and again after growing the cells for an additional 3.5 hours at 36˚C. The cells were then fixed and stained with DAPI and methylene blue. Representative images are shown. The experiment was performed in duplicate. Scale bar, 5 µm. (D) The number of nuclei per cell (top), and the percentage of septated cells (middle) and lysed cells (bottom) were quantified at 36°C from the same experiments as in C. N≥200 cells of each genotype. (E) Ribbon diagram of a structural model of *S. pombe* Mob1 using AlphaFold3 (Abramson et al., 2024). The positions of the N- and C-termini and the positions of the mutated residues in the *mob1* alleles are indicated. (F) Schematic of *sid2* gene product (drawn to scale). Numbers indicate amino acid position (top). Ribbon diagram of a structural model of *S. pombe* Mob1 bound to the Sid2 regulatory region and kinase domain using AF3. Mob1 is in magenta, the Sid2 regulatory region is in green, and the Sid2 kinase domain is in cyan (bottom).

## Description

Cytokinesis in *Schizosaccharomyces pombe* requires the construction of an actin- and myosin-based cytokinetic ring (CR) that is coupled to the formation of the division septum (Cheffings et al., 2016; Glotzer, 2017; Mangione & Gould, 2019). The septation initiation network (SIN) is a signaling cascade that promotes CR formation, CR constriction and septum formation [reviewed in (Cullati & Gould, 2019; Simanis, 2015; Xiao & Dong, 2021)]. The SIN is assembled at the mitotic spindle pole body and initiated by the GTPase Spg1 (Schmidt et al., 1997; Sohrmann et al., 1998). Spg1 activates the kinase Cdc7 which in turn activates the Sid1 kinase (Fankhauser & Simanis, 1993; Schmidt et al., 1997). Downstream of Sid1 is the Sid2 kinase and its obligate binding partner, Mob1 . The Sid2- Mob1 complex is the sole SIN component that translocates from the spindle pole body to the CR curing cytokinesis (Guertin et al., 2000; M. C. Hou et al., 2000; Salimova et al., 2000; Sparks et al., 1999). At the CR Sid2- Mob1 phosphorylates and regulates key substrates such as formin Cdc12 , anillin-like Mid1 , and Cdc14 family phosphatase Clp1 , important for various cell division cycle steps (Bohnert et al., 2013; Chen et al., 2008; Sparks et al., 1999; Willet et al., 2019). SIN signaling is inhibited by the Cdc16- Byr4 two-component GAP for Spg1 (Furge et al., 1998; Minet et al., 1979).

Here, we examine a set of temperature-sensitive strains of *mob1* ( *mob1-N1* , *mob1-M17* , *mob1-J2* and *mob1-* R4) that were isolated from a genetic screen designed to identify suppressors of *cdc7-A20 cdc16∆* cold-sensitivity (Salimova et al., 2000). The isolated alleles were within the same complementation group and rescued by expression of *mob1 ^+^* from a plasmid (Salimova et al., 2000). Further, *mob1-R4* was found to suppress *cdc16∆* in the absence of *cdc7-A20* and all *mob1* alleles also rescued *byr4∆* lethality (Salimova et al., 2000). An additional temperature sensitive allele, *mob1-1* , was designed based on a *Saccharomyces cerevisiae* allele of the orthologous protein (Hou et al., 2000; Luca and Winey, 1998). Mob1 was found to bind the Sid2 kinase and be essential for its localization and activity (Hou et al., 2000; Salimova et al., 2000). These results established Mob1 as a positive regulator of SIN signaling.

To determine what mutations were present in the *mob1* alleles, the open reading frame was amplified from each strain and sequenced. *mob1-N1* , *mob1-M17* and *mob1-J2* encoded the same mutation causing the change of histidine 159 to tyrosine ( Figure 1A ). *mob1-R4* encoded a substitution at residue 155 of arginine to cystine ( Figure 1A ). *mob1-1* encodes the I55P and A56I mutations (Hou et al., 2000). Thus, there are now three distinct temperature-sensitive *mob1* mutant alleles with known mutations.

We further characterized *mob1-N1* and *mob1-R4* and compared them to *mob1-1* , eliminating the duplicative *mob1-M17* and *mob1-J2* alleles from further analysis. The range of temperature-sensitivity was determined for each strain by spotting at a variety of temperatures. *mob1-1* grew less well than wildtype at 32°C and 36°C and *mob1-N1* grew less well than wildtype at 36°C ( Figure 1B ). *mob1-R4* grew slightly less well than wildtype at both high (32°C and 36°C) and low (25°C) temperatures ( Figure 1B ). Next, to visualize cell phenotypes, we fixed and stained cells to mark nuclei and septa after they were grown at 25°C and also, after shifting to 36˚C for 3.5 hours. All *mob1* alleles looked similar to wildtype at 25°C ( Figure 1C ). At 36°C, *mob1-1* cells were multinucleated with no septum present ( Figure 1C ), as has been previously reported (Hou et al., 2000). *mob1-N1* and *mob1-R4* displayed some binucleate cells with no septum ( Figure 1C- D), consistent with previous results for *mob1-R4* (Salimova et al., 2000). A percentage of lysed cells were also detected for *mob1-N1* and *mob1-R4* ( Figure 1C- D). Due to the observed cytokinesis defects at high temperatures, we conclude that all *mob1* alleles are likely loss-of-function alleles and *mob1-1* is the most severe temperature sensitive allele.

Mob1 is part of a family of small proteins that are allosteric activators of kinases (Duhart and Raftery, 2020). We mapped the amino acids that were changed in the *mob1* alleles onto the AlphaFold3 predicted protein structure (Abramson et al., 2024). We found that the mutation sites are not clustered within a specific region of the Mob1 protein and are within regions that mediate intramolecular interactions thus, mutation of these residues may result in a less stable protein ( Figure 1E ).

Mob1 family members typically bind kinase partners within the N-terminal regulatory region just upstream of the catalytic domain (Duhart and Raftery, 2020). In accord, co-immunoprecipitation experiments determined that Mob1 bound a fragment of Sid2 containing residues 101-207, which is just upstream of the kinase domain (Hou et al., 2004). To better understand the interaction, we modelled the Sid2- Mob1 interaction using AF3. It was predicted that a set of Sid2 ɑ-helices formed by residues 125-200 bound Mob1 within the regulatory region ( Figure 1F ). Further, none of the amino acids mutated in the *mob1* alleles were involved in the predicted Mob1- Sid2 interaction interface.

## Methods

Yeast methods

*S. pombe* strains were grown in yeast extract (YE) and standard *S. pombe* mating, sporulation, and tetrad dissection techniques were used to construct new strains (Moreno et al., 1991).

Molecular biology methods

The *mob1* alleles were amplified using an oligonucleotide 20 bp upstream of the start site (GAGTTTACCTCCCATTTCCTGTTCC) and 50 bp downstream of the stop codon (GACGATGAGTGGAAGGTTGG) (Integrated DNA technologies). The PCR products were sequenced by Plasmidsaurus (Eugene, OR) using Oxford Nanopore Technology with custom analysis and annotation.

Microscopy and image analysis

Strains for fixed-cell imaging experiments were grown at 25°C in YE and then shifted to 36°C for 3.5 hours. Cells were fixed with 70% ethanol for DAPI and methylene blue (MB) staining as described previously (Roberts-Galbraith et al., 2009). Images were acquired using a Zeiss Axio Observer inverted epifluorescence microscope with Zeiss 63× oil (1.46 NA) objective and captured using Zeiss ZEN 3.0 (Blue edition) software. A singular medial Z slice was obtained. All images were further processed using ImageJ (Schindelin et al., 2012).

AlphaFold3 structural prediction

Protein structure predictions were generated with the AlphaFold3 server (Abramson et al., 2024) and visualized using the PyMOL molecular graphics system (version 3.0, Schrodinger, LLC).

## Reagents

**Table:** 

**Strain**	**Genotype**	**Source**
KGY246	*ade6-M210 leu1-32 ura4-D18* *h ^-^*	Lab stock
KGY4464	* mob1-N1 leu1-32 h ^-^*	Salimova et al., 2000
KGY3220	* mob1-R4 ade6-M21X ura4-D18 leu1-32 h ^-^*	Salimova et al., 2000
KGY1428-2	* mob1-1 ade6-M21X leu1-32 ura4D-18 h ^+^*	Hou et al., 2000
KGY4465	* mob1-M17 leu1-32 h ^-^*	Salimova et al., 2000
KGY4466	* mob1-J2 leu1-32 h ^-^*	Salimova et al., 2000

## References

[R1] Abramson Josh, Adler Jonas, Dunger Jack, Evans Richard, Green Tim, Pritzel Alexander, Ronneberger Olaf, Willmore Lindsay, Ballard Andrew J., Bambrick Joshua, Bodenstein Sebastian W., Evans David A., Hung Chia-Chun, O’Neill Michael, Reiman David, Tunyasuvunakool Kathryn, Wu Zachary, Žemgulytė Akvilė, Arvaniti Eirini, Beattie Charles, Bertolli Ottavia, Bridgland Alex, Cherepanov Alexey, Congreve Miles, Cowen-Rivers Alexander I., Cowie Andrew, Figurnov Michael, Fuchs Fabian B., Gladman Hannah, Jain Rishub, Khan Yousuf A., Low Caroline M. R., Perlin Kuba, Potapenko Anna, Savy Pascal, Singh Sukhdeep, Stecula Adrian, Thillaisundaram Ashok, Tong Catherine, Yakneen Sergei, Zhong Ellen D., Zielinski Michal, Žídek Augustin, Bapst Victor, Kohli Pushmeet, Jaderberg Max, Hassabis Demis, Jumper John M. (2024). Accurate structure prediction of biomolecular interactions with AlphaFold 3. Nature.

[R2] Bohnert K. Adam, Grzegorzewska Agnieszka P., Willet Alaina H., Vander Kooi Craig W., Kovar David R., Gould Kathleen L. (2013). SIN-dependent phosphoinhibition of formin multimerization controls fission yeast cytokinesis. Genes & Development.

[R3] Cheffings Thomas H., Burroughs Nigel J., Balasubramanian Mohan K. (2016). Actomyosin Ring Formation and Tension Generation in Eukaryotic Cytokinesis. Current Biology.

[R4] Chen Chun-Ti, Feoktistova Anna, Chen Jun-Song, Shim Young-Sam, Clifford Dawn M., Gould Kathleen L., McCollum Dannel (2008). The SIN Kinase Sid2 Regulates Cytoplasmic Retention of the S. pombe Cdc14-like Phosphatase Clp1. Current Biology.

[R5] Cullati Sierra N., Gould Kathleen L. (2019). Spatiotemporal regulation of the Dma1-mediated mitotic checkpoint coordinates mitosis with cytokinesis. Current Genetics.

[R6] Duhart Juan Carlos, Raftery Laurel A. (2020). Mob Family Proteins: Regulatory Partners in Hippo and Hippo-Like Intracellular Signaling Pathways. Frontiers in Cell and Developmental Biology.

[R7] Fankhauser C, Homans SW, Thomas Oates JE, Mc Conville MJ, Desponds C, Conzelmann A, Ferguson MA. 1993. Structures of glycosylphosphatidylinositol membrane anchors from Saccharomyces cerevisiae.. The Journal of biological chemistry. 268: 26365. 8253761

[R8] Furge Kyle A., Wong Kelvin, Armstrong John, Balasubramanian Mohan, Albright Charles F. (1998). Byr4 and Cdc16 form a two-component GTPase-activating protein for the Spg1 GTPase that controls septation in fission yeast. Current Biology.

[R9] Glotzer Michael (2016). Cytokinesis in Metazoa and Fungi. Cold Spring Harbor Perspectives in Biology.

[R10] Guertin D. A. (2000). The role of the Sid1p kinase and Cdc14p in regulating the onset of cytokinesis in fission yeast. The EMBO Journal.

[R11] Hou Ming-Chin, Salek Jeffrey, McCollum Dannel (2000). Mob1p interacts with the Sid2p kinase and is required for cytokinesis in fission yeast. Current Biology.

[R12] Hou Ming-Chin, Guertin David A., McCollum Dannel (2004). Initiation of Cytokinesis Is Controlled through Multiple Modes of Regulation of the Sid2p-Mob1p Kinase Complex. Molecular and Cellular Biology.

[R13] Luca Francis C., Winey Mark (1998). *MOB1*
, an Essential Yeast Gene Required for Completion of Mitosis and Maintenance of Ploidy. Molecular Biology of the Cell.

[R14] Mangione MariaSanta C., Snider Chloe E., Gould Kathleen L. (2019). The intrinsically disordered region of the cytokinetic F-BAR protein Cdc15 performs a unique essential function in maintenance of cytokinetic ring integrity. Molecular Biology of the Cell.

[R15] Minet M, Nurse P, Thuriaux P, Mitchison J M (1979). Uncontrolled septation in a cell division cycle mutant of the fission yeast Schizosaccharomyces pombe. Journal of Bacteriology.

[R16] Moreno Sergio, Klar Amar, Nurse Paul (1991). [56] Molecular genetic analysis of fission yeast Schizosaccharomyces pombe. Guide to Yeast Genetics and Molecular Biology.

[R17] Roberts-Galbraith Rachel H., Chen Jun-Song, Wang Jianqiu, Gould Kathleen L. (2009). The SH3 domains of two PCH family members cooperate in assembly of the
*Schizosaccharomyces pombe*
contractile ring. The Journal of Cell Biology.

[R18] Salimova Ekaterina, Sohrmann Marc, Fournier Nadine, Simanis Viesturs (2000). The S. pombe orthologue of the S. cerevisiae mob1 gene is essential and functions in signalling the onset of septum formation. Journal of Cell Science.

[R19] Schindelin Johannes, Arganda-Carreras Ignacio, Frise Erwin, Kaynig Verena, Longair Mark, Pietzsch Tobias, Preibisch Stephan, Rueden Curtis, Saalfeld Stephan, Schmid Benjamin, Tinevez Jean-Yves, White Daniel James, Hartenstein Volker, Eliceiri Kevin, Tomancak Pavel, Cardona Albert (2012). Fiji: an open-source platform for biological-image analysis. Nature Methods.

[R20] Schmidt S, Sohrmann M, Hofmann K, Woollard A, Simanis V (1997). The Spg1p GTPase is an essential, dosage-dependent inducer of septum formation in Schizosaccharomyces pombe.. Genes & Development.

[R21] Simanis Viesturs (2015). Pombe's thirteen – control of fission yeast cell division by the septation initiation network. Journal of Cell Science.

[R22] Sohrmann Marc, Schmidt Susanne, Hagan Iain, Simanis Viesturs (1998). Asymmetric segregation on spindle poles of the
*Schizosaccharomyces pombe*
septum-inducing protein kinase Cdc7p. Genes & Development.

[R23] Sparks CA, Morphew M, Mc Collum D. 1999. Sid2p, a spindle pole body kinase that regulates the onset of cytokinesis.. The Journal of cell biology. 146: 777. 10.1083/jcb.146.4.777 PMC2156147 10459013

[R24] Willet Alaina H., DeWitt Ashley K., Beckley Janel R., Clifford Dawn M., Gould Kathleen L. (2019). NDR Kinase Sid2 Drives Anillin-like Mid1 from the Membrane to Promote Cytokinesis and Medial Division Site Placement. Current Biology.

[R25] Xiao Yi, Dong Jixin (2021). The Hippo Signaling Pathway in Cancer: A Cell Cycle Perspective. Cancers.

